# Supplementary material for: Feasibility of a Novel Therapist-Assisted Feedback System for Gait Training in Parkinson’s Disease
Source: Sensors (Basel). 2022 Dec 23;23(1):128. doi: 10.3390/s23010128 (PMC9823339; doi:10.3390/s23010128)
Supplement: Supplementary file 1 [file sensors-23-00128-s001.zip › sensors-2037297-supplementary.pdf]

Supplementary Figure S1.

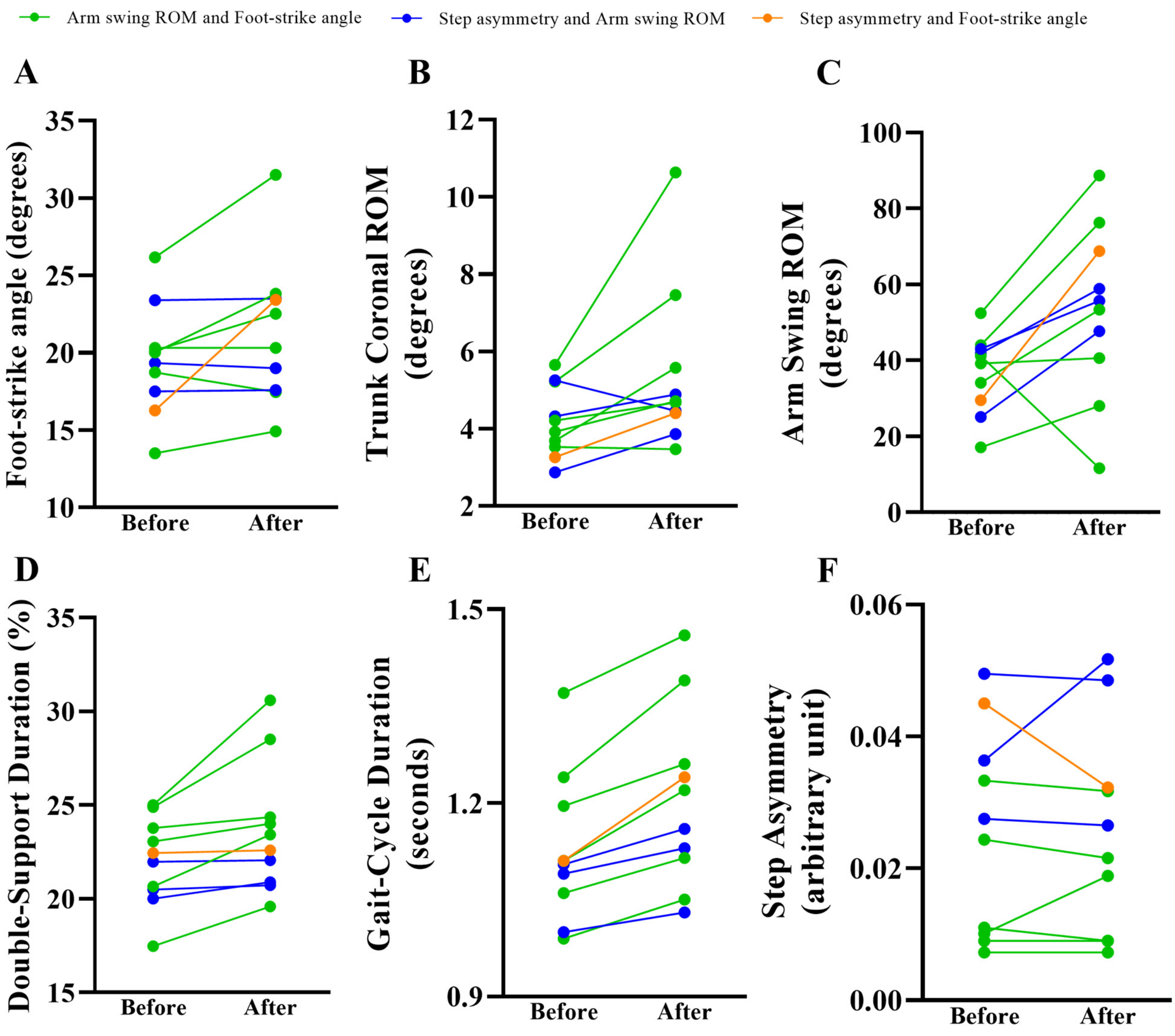

Supplementary Figure S1. Individual values for the foot-strike angle (**A**), trunk coronal rangeof-motion (ROM) (**B**), arm swing range-of-motion (**C**), double-support duration (**D**), gait-cycle duration (**E**), and step asymmetry (**F**) before and after one session of treadmill gait training with Mobility Rehab in 10 people with Parkinson's disease. Colours represent gait metrics selected to train people with PD.
